# Influence of distal radius fractures involving the intermediate column on forearm rotation

**DOI:** 10.1186/s13018-019-1155-4

**Published:** 2019-04-24

**Authors:** Bingshan Yan, Yanchao Chen, Wangping Yin

**Affiliations:** Department of Orthopedic Surgery, Jinshan Hospital, Fudan University, Shanghai, 201508 People’s Republic of China

**Keywords:** Distal radius fracture, Triangular fibrocartilage complex, A three-column theory, The intermediate column

## Abstract

**Background:**

The objective of the study was to compare the radiologic and clinical outcome of patients with distal radius fractures involving the intermediate column and distal radial metaphyseal fractures treated conservatively.

**Methods:**

Two cohorts of 52 matched patients with distal radius fractures treated conservatively, one with a fracture involving the intermediate column and the other with no intermediate column fracture, were retrospectively analyzed by examining the data. Patients were matched for age, sex, fracture side, and AO fracture type. The two groups were analyzed for differences in wrist motion; grip strength; Gartland and Werley score; Disabilities of the Arm, Shoulder and Hand (DASH) score; and visual analogue scale (VAS) score at 12 months. The differences in continuous variables were compared using the paired *t* test. Linear regression analyses or Pearson correlation analyses were used to evaluate the associations of radiological parameters with clinical outcomes.

**Results:**

The analysis showed significant differences in the range of motion (ROM) for pronation (*p* = 0.000) and supination (*p* = 0.008) in the paired groups. There was a significant difference in DASH scores (*p* = 0.024) in the paired groups. Using Pearson correlation analysis, negative correlations (*r* = − 0.360, *p* = 0.000) were observed between articular step-off and ROM for pronation. Linear regression analyses also indicated that ROM for pronation had negative relationships (*β* = − 6.327, *p* = 0.001) with articular step-off.

**Conclusions:**

Distal radius fractures involving the intermediate column had an adverse effect on forearm rotation after distal radius fractures treated conservatively.

## Background

Distal radius fractures are one of the most common injuries of all extremity fractures, with 41–50% involving articular surfaces of the distal radius end [1]. Currently, there are many classifications of distal radius fractures. Each fracture classification has its own advantages. Rikli and Regazzoni proposed a three-column biomechanical construction forming the distal radius and the distal ulna to guide surgical fixation [2]. In their theory, the intermediate column consists of the lunate fossa and the sigmoid notch, which is vital to transmit the load in the wrist. Anatomically, the sigmoid notch of the distal radius, where the triangular fibrocartilage complex (TFCC) origins from, articulates with the convex ulnar head [3]. This means that any incongruency of the sigmoid notch and the ulnar head may lead to pain or dysfunction of the distal radioulnar joint (DRUJ) [4].

Many studies have reported that distal radius fractures need anatomical reconstruction, stable fixation, and early function as any other intra-articular fractures [5], especially in fractures of the intermediate column of the wrist. It is widely believed that there is a close correlation between anatomical results and functional outcomes [2]. But, as far as we know, there is little direct evidence of the correlation between the fractures of intermediate column and the wrist outcome. Thus, we designed a 1:1 matched retrospective case-control study, which analyzed clinical data of 52 distal radius fractures involving the intermediate column (C1, C2) using the AO classification matched with 52 cases with distal radial metaphyseal fractures (A2, A3). All cases were treated conservatively. Our null hypothesis was that fractures of the intermediate column of the wrist would affect wrist function, especially forearm rotation.

## Materials and methods

This investigation got our hospital review board approval. Inclusion criteria were as follows: the patient with distal radius fractures classified as distal radius fractures involving the intermediate column fracture (C1, C2) or not (A2, A3) and no history of wrist medical morbidities. Distal radius fractures involving the intermediate column have three types: only the radiocarpal joint involved (rarely), involving both the radiocarpal joint and the radioulnar joint, or only the radioulnar joint involved. Exclusion criteria included patients above the age of 70 years, distal radius fractures treated operatively, refusal to return for follow-up evaluation, or skeletally immature patients. Open fractures or multiple fractures were also excluded. The study used a 1:1 matched case-control design. Distal radius fractures were classified according to the AO/ASIF system. Two groups of 104 patients were matched on the following factor: sex, age within 5-year groups, fracture side, and fracture type. The case group was distal radius fractures involving the intermediate column fracture (C1, C2), and the control group was extra-articular fracture of the distal radius (A2, A3). Type A2 matched type C1, as well as type A3 matched type C2. All patients were undergone closed reduction and casting.

Patients revisited for clinical assessments at 3 and 12 months. The primary parameter of clinical assessments for the injured and uninjured sides was active wrist and forearm ROM (extension, flexion, radial deviation, ulnar deviation, supination, and pronation). Active wrist and forearm ROMs were evaluated following a standard guideline [6]. An independent author performed every ROM measure three times. The mean of three measured values was adopted for the analysis. The data we recorded was the percentage of the affected side compared to the contralateral side. Grip strength was measured and recorded in the same way.

X-rays were used to evaluate the alignment of the distal part of the radius on posteroanterior and lateral radiographs using the picture archiving and communication system. X-rays taken at immediate post-reduction and at the last follow-up were adopted to measure radiographic parameters [7]. Radiographic parameters included radial inclination, ulnar inclination, palmar tilt, and articular step-off. In this study, we defined the articular step-off as an independent factor and used it to distinguish whether there is a fracture involving the intermediate column. All X-rays were also measured three times by an attending orthopedic surgeon. A mean of measurements was used for the analysis. At the last follow-up, grip strength, Disabilities of the Arm, Shoulder and Hand (DASH) scores, Gartland and Werley scores, and VAS scores were used to evaluate wrist function [8].

## Statistical analysis

Continuous variables included age, ROM, articular step-off, grip strength, VAS, and DASH. The mean, standard deviation, and 95% confidence intervals of continuous variables were calculated. The differences in continuous variables were compared using the paired *t* test or Wilcoxon signed-rank test. Categorical variables were evaluated using the chi-squared test or Fisher exact test. Pearson correlation analysis was performed to evaluate the associations of articular step-off and ROM. Linear regression analyses were used to evaluate the associations of radiological parameters with clinical outcomes. *p* values less than 0.05 were considered statistically significant. SPSS Statistical Software Package for Windows (version 23.0) was used for the statistical analysis in a personal computer.

## Results

Between January 2014 and October 2017, 104 patients with a mean age of 52.5 ± 9.3 years (range, 25–66) satisfied the inclusion criteria. There were 42 males and 62 females. Eighteen patients had a type A2 fracture, 34 type A3, 18 type C1, and 34 type C2. Fifty-four patients were accompanied by ulnar styloid fractures. The mean follow-up time was 15 months (range, 12 to 24 months). Using a paired Student’s *t* test for continuous variables and the paired chi-squared test for categorical variables, there were no statistically significant differences between two matched cohorts on the basis of age, sex, fracture side, and ulnar styloid fracture (Table 1).

**Table 1:** Descriptive data of the matched cohorts

	Case group	Control group	*p* value
Sex			0.781
Male	21	21	
Female	31	31	
Age (years)	52.7 ± 9.3	52.4 ± 9.4	0.454
Fracture side			0.163
Left	24	24	
Right	28	28	
Ulnar styloid fracture			1.00
Yes	27	27	
No	25	25	

Grip strength, DASH, VAS, Gartland and Werley scores, radiographic parameters, and wrist and forearm active ROMs at the final follow-up for the two cohorts are summarized in Table 2. There were significant differences in ROM for pronation (*p* = 0.000) and supination (*p* = 0.008) between the matched cohorts. We also observed a significant difference in DASH scores (*p* = 0.024) between the matched cohorts. No significant differences were found in any other variable. We found statistically negative correlations (*r* = − 0.360, *p* = 0.000) between articular step-off and ROM for pronation. In a stepwise forward regression, the results also revealed only ROM for pronation had significant relationships (*β* = − 6.327, *p* = 0.001) with articular step-off. At 1 year, the mean value of intra-articular step-off was 2.0 mm in the case cohort. With respect to the volar angulation, radial inclination of the distal radial fracture, there were no significant differences between the matched cohorts at the final follow-up (Table 2).

**Table 2:** Radiographic and clinical outcomes in the matched cohorts

Parameters	Case group	Control group	*p*
Volar tilt (degrees)	7.5 ± 4.9	7.4 ± 4.2	0.672
Radial inclination (degrees)	20.5 ± 3.6	20.1 ± 3.1	0.153
Intra-articular step-off (mm)	2.0 ± 0.9		
Range of motion (% of the contralateral side)			
Flexion	73.1 ± 9.6	73.9 ± 8.9	0.450
Extension	73.8 ± 7.5	73.1 ± 8.5	0.586
Pronation	77.0 ± 7.8	82.8 ± 7.3	0.000
Supination	72.0 ± 7.4	74.9 ± 8.7	0.008
Radial deviation	81.9 ± 6.3	83.2 ± 7.1	0.328
Ulnar deviation	78.6 ± 6.4	77.2 ± 4.9	0.244
Ulnar styloid fractures	27	27	1.000
Grip strength (% of the contralateral side)	86.2 ± 5.4	87.7 ± 4.8	0.175
DASH	13.0 ± 5.8	11.0 ± 4.2	0.024
VAS	1.0 ± 1.0	1.0 ± 1.00	1.000
Gartland and Werley scores	3.71 ± 1.52	3.63±1.59	0.794

## Discussion

The purpose of this study was to analyze the clinical and radiologic outcomes of distal radius fractures involving the intermediate column (C1, C2) and distal radius fractures (A1, A2) treated conservatively. The null hypothesis was that distal radius fractures involving the intermediate column have a significantly poorer radiologic and clinical outcome. The analysis of the data supported the hypothesis. In this study, the ROM for pronation and supination revealed significant differences between the matched cohorts. It also suggested that distal radius fractures involving the intermediate column had a worse result in DASH scores. So far, there were few reports specialized on fractures of the intermediate column. This type of fracture was included in some other researches. From the view of the three-column theory, the die-punch fracture should be classified as the intermediate column fracture. In a study of investigating results of AO type C fractures of distal radius treated by volar locking plate, LV et al. reported that die-punch fractures had poorer clinical outcomes at an early postoperative period [1].

As mentioned before, articular step-off represented a fracture involving the intermediate column in the study. We found statistically negative correlations between articular step-off and ROM for pronation in the matched cohorts. This meant that the more the displacement of articular step-off, the worse the rotation function of the forearm. Our result is not contradictory to the previously published study: Swart et al. found after operatively treated distal radius fractures, supination improved more quickly, usually within the first 3 to 6 months [9].

There are many plausible explanations for these results. In view of anatomy, the sigmoid notch of the radius constitutes the intermediate column. The sigmoid notch which serves as an anchor for the TFCC probably plays a role in DRUJ stability [3]. It makes sense: when the displaced fracture fragment involving the sigmoid notch apparently changed the tension of the TFCC, there would produce a rotation dysfunction of the forearm. Moreover, the displaced fragment could cause articular incongruity of the DRUJ and jeopardize the rotation of the forearm [4, 10]. Ishikawa et al. reported that the malposition of the ulnar head might result in restricted pronation because of the change of the tension of the surrounding soft tissues [11]. Adams confirmed that displaced distal radial fractures could place immense stress on the TFCC, which may result in a decreased pronation and/or supination [12]. Delclaux et al. found the patient with distal radius fracture malunion basically improved their wrist functions of pronation/supination after the corrective osteotomy [13].

Besides fracture morphology and fracture displacement, the presence of ulnar styloid fractures is supposed to be a susceptibility factor for DRUJ instability associated with distal radius fractures [14]. Some authors have suggested that ulnar styloid fractures accompanied by distal radius fracture are associated with wrist outcomes and increase the risk of DRUJ instability [15, 16]. However, more and more researches have supported that ulnar styloid fracture does not appear to influence on wrist function or outcome in patients with distal radial fractures [17–19]. Due to the ongoing debate in ulnar styloid fracture, we controlled this possible confounder between two groups. There was no significant difference (*p* = 1.00) in the ulnar styloid fracture.

Chung et al. demonstrated that after operatively treated distal radius fractures, age was significantly related to long-term outcomes. They showed the older patients were vulnerable to have poorer clinical outcomes [20]. Larouche et al. reported that patients with distal radius fractures older than 55 years did not gain a better outcome in the surgical group as compared with the conservative group [21]. Hohmann et al. supported that elderly patients with minor malunion of the distal radius had no adverse effect on patient-perceived outcomes regardless of whether these fractures were treated operatively or conservatively [22]. The above findings indicated that age was vital to influence patient-perceived outcomes after the treatment of distal radius fractures. However, our results showed there was no statistical difference between two matched cohorts in age.

This study has several potential shortcomings. First, this is a retrospective study, which has its drawbacks such as selection bias and limited clinical data. Second, due to lack of a very good or excellent inter- and intra-observer reproducibility in the existing classifications of distal radius fractures [23], AO subclassifications based on X-ray and/or CT scans may also have inconsistencies, leading to changes in our conclusion. Ma et al. described that X-ray results might be false negative in diagnosis of die-punch fracture. The X-ray-missed diagnosis rate was 11.1%, and the misdiagnosis rate was 15.6% [24]. Third, the issue of how to avoid variability in ROM measurement still cannot be completely solved. Hohmann et al. reported there was accurate, valid, and reliable use of a goniometer according to a standardized protocol advised by the American Society for Hand Therapists [22]. Therefore, we used the goniometer to measure wrist ROM following the standard guideline to reduce variability. And we supported that clinical examination comparing between the injured and uninjured contralateral sides was a reasonable way to reduce individual differences in the assessment of objective indicators of wrist function after distal radius fractures.

## Conclusions

The results prove that fractures of the intermediate column play an important role in determining restricted forearm rotation after distal radius fractures treated conservatively. The data also suggest that patients with distal radius fractures involving the intermediate column have lower DASH scores. Based on these findings, we think fractures involving the intermediate column of the distal end deserve more attentions from the surgeon. However, there still have several risks of bias in this study. A further investigation is needed to better understand the complex relationship between anatomy, function, and radiologic parameters in patients with fractures involving the intermediate column of the distal end.

## Abbreviations

DASH: Disabilities of the Arm, Shoulder and Hand
DRUJ: Distal radioulnar joint
ROM: Range of motion
TFCC: Triangular fibrocartilage complex
VAS: Visual analogue scale
